# Dietary compliance and its determinants among type 2 diabetes patients in Tamale Metropolis, Ghana

**DOI:** 10.1186/s41043-024-00588-2

**Published:** 2024-06-19

**Authors:** Ambrose Atosona, Lisa Boakye Yiadom, Barichisu Alhassan, Hamida Kelli, Patience Kanyiri Gaa, Gabriel Libienuo Sowley Kalog

**Affiliations:** Department of Nutritional Sciences, School of Allied Health Sciences, University for Development Studies, Tamale, Ghana

**Keywords:** Dietary compliance, Type 2 diabetes, Nutrition, Ghana

## Abstract

**Background:**

The prevalence of type 2 diabetes is currently high and still rising, predominantly in developing countries including Ghana. Type 2 diabetes patients’ adherence to recommended diet is shown to improve their health outcomes. This study investigated dietary compliance and its determinants among type 2 diabetes patients in Tamale Metropolis, Ghana.

**Methods:**

This study employed analytical cross-sectional study design and involved 343 participants selected through systematic random sampling from the outpatient diabetes clinics of Tamale Teaching Hospital, Tamale West Hospital and Tamale Central Hospital. A semi-structured questionnaire was used to document participants’ socio-demographic, lifestyle and clinical characteristics. The modified Morisky dietary adherence scale was used to assess dietary compliance. Bivariate and multivariate analyses were performed to determine the predictors of dietary compliance.

**Results:**

The mean age of participants was 56.4 ± 15.7 years. More than half of the participants (62.4%) were females. The participants had a higher compliance status (70.6%). The study also revealed that, as a patient’s age increases, compliance decreases [Adjusted odd ratio (AOR): 0.96, 95%, Confidence interval (CI) 0.94–0.99, *P* = 0.002]. Regarding educational status, patients who completed JHS/Middle School [AOR: 2.458, 95% CI 1.019–5.928, *P* = 0.045] and SHS/Vocational School [AOR: 2.73, 95% CI 1.08–6.91, *P* = 0.035] were more likely to comply with dietary recommendations compared to those with no formal education.

**Conclusion:**

The rate of dietary compliance was high among the study participants. Age and educational status of participants significantly influenced their dietary compliance, suggesting that these factors should be taken into account when developing strategies to enhance dietary adherence.

## Background

The global prevalence of diabetes is high and still increasing particularly in low- and middle-income nations. The global prevalence in 2021 stood at 10.5%, expected to rise to 11.3% by 2030 and 12.2% by 2045 [1]. Africa is estimated to have 23.6 million adults living with diabetes with a regional prevalence of 4.5% [1]. The prevalence of diabetes among adults in Ghana is 6.5% [2]. Type 2 diabetes accounts for over 90% of all diabetes worldwide and is characterised by high blood glucose resulting from the body's inability to use insulin [3]. Increased thirst, frequent urination, fatigue, blurred vision, slow healing sores, frequent infections, weight loss are some of the signs and symptoms of type 2 diabetes [3]. Type 2 diabetes is mainly caused by overweight, obesity and physical inactivity [3]. Over time, hyperglycaemia results in the damage of the heart, blood vessels, eyes, kidneys and nerves [4]. These complications lead to human suffering, economic burden and mortality [5]. It is estimated that about 6.7 million global deaths are attributed to diabetes [1].

Promoting a lifestyle that includes a healthy diet is the cornerstone of type 2 diabetes management [1]. People with type 2 diabetes are advised to consume high fibre and low-glycaemic index foods (whole grains, fruits, vegetables, legumes, nuts, fish, monounsaturated fats etc.) and avoid sugar, sweets and sweetened beverages [6, 7]. Additionally, they are encouraged to abstain from tobacco use and limit the intake of alcohol [8]. Sticking to these dietary recommendations has been demonstrated to improve patient’s glycaemic control, health outcomes and well-being [9, 10]. However, most diabetes patients have difficulty following dietary recommendations despite knowing the importance of diet in diabetes management [11]. According to Ranasinghe et al*.* [11]*,* the difficulty in applying dietary recommendations in real life are greatly influenced by social factors. Prevalence rates of dietary adherence among type 2 diabetes patients in southern Ghana ranges from 26.7 to 69% [10, 12]. Also, prevalence rates of 41.1%, 60%, 15.7% and 21% have been reported in Ethiopia [13], Kenya[14], Nepal [15] and Yemen [16] respectively. Potential associated factors of dietary compliance among type 2 diabetes patients include duration of diabetes, diabetic nutrition education, monthly income, educational level, dietary knowledge and employment status [13, 15–17].

Despite the crucial role diet plays in the management of type 2 diabetes, information on the level and predictors of compliance to dietary recommendations among type 2 diabetes patients in Northern Ghana, particularly Tamale Metropolis is limited. This study therefore sought to investigate the level of dietary compliance and its predictors among type 2 diabetes patients in the Tamale Metropolis, Ghana.

## Methods

### Study design, area, population and sampling technique

An analytical cross-sectional study design was employed and involved type 2 diabetes patients attending the outpatient diabetic clinics of the Tamale Teaching Hospital, Tamale West Hospital and Tamale Central Hospital in the Tamale Metropolis. Patients with type 2 diabetes diagnosed according to WHO criteria [8] (fasting plasma glucose ≥ 7.0 mmol/L and/or 2 h postprandial plasma glucose or random plasma glucose ≥ 11.1 mmol/L), who were 18 years and above and consented to participate in the study were enrolled. Pregnant, critically ill and mentally unstable patients were excluded. Systematic random sampling technique was used to select study subjects.

### Sample size

The sample size for the study was calculated using Yamane formula; n=$\frac{N}{1+N{e}^{2}}$ [18]

Where n is the required sample size.

N is the total population. Total number of type 2 diabetes patients in the study hospitals is about 1800 [19].

E is the acceptable sampling error (0.05) at 95% Confidence Interval.

By substitution:

$$\begin{aligned} {\text{n}} & = \frac{1800}{{1 + 1800\left( {0.05} \right)^{2} }} \\ {\text{n}} & = { 327} \\ \end{aligned}$$

Non-response rate of 5% = 327 × 0.05 = 16.35≈16.

Hence, the sample size for the study was 327 + 16 = 343 participants.

### Data collection

Pretested semi-structured questionnaire was used to document participants’ information on socio-demographic (education, gender, marital status, religion, income level etc.), lifestyle and clinical characteristics. The modified morisky dietary adherence scale adopted from a previous study in Ethiopia [17] was used to assess dietary compliance. The questionnaire consist of 10 questions. Each question has two response options (Yes = 1 and No = 0). The scores for each participant were summed and average score computed. A score below the average value was deemed adherent. The tool has a reliability of 0.74 [17].

Diabetes-related nutrition knowledge was assessed using a validated questionnaire [20]. The questionnaire consist of 12 questions. A correct answer to each question was assigned 5 points while a wrong answer was assigned 0. The scores were summed and converted to 100%. A score of 50% or more was considered as good knowledge.

The body mass index was used to assess nutritional status of participants. Height (m) was measured without shoes using microtoise (Seca, Germany) and weight (kg) measured, whilst each participant was in light clothing using an electronic scale (Seca, Germany). The body mass index was calculated by dividing the weight by the square of the height and was classified as underweight (> 18.49 kgm^−2^), normal (18.5–24.99 kgm^−2^), overweight (25–29.99 kgm^−2^), Obese (≥ 30 kgm^−2^) according to the World Health Organization criteria [21].

### Data analysis

Analysis of all data was performed using SPSS statistical software version 25 (IBM, USA). Continuous variables were presented as means and standard deviations while categorical variables were presented as frequencies. Bivariate analysis was done using chi-square test. Variables with *P*-value < 0.25 [22] in the bivariate analysis were considered for multivariate binary logistic regression analysis to identify predictors of dietary compliance. *P*-value less than 0.05 was considered statistically significant at 2 tailed tests.

## Results

### Participants’ characteristics and dietary compliance

Table 1 depicts the socio-demographic characteristics of the participants. The study revealed that about 37.6% and 62.4% of the participants were males and females, respectively, with a mean age of 56.4 ± 15.7 years. Majority of the participants were Dagombas and Gonjas representing 45.5% and 17.2% respectively. Also, most (35.9%) of the respondents had no formal education, and were self- employed (43.7%). More than half of them (51%) earned less than GHȻ500 per month. Concerning lifestyle and clinical characteristics, majority of the participants never smoked (84.8%) and never took alcohol (75.8%). Also, most of the participants had diabetes duration less than 5 years (41.7%) and good nutritional knowledge (46.6%) (Table 2). Regarding dietary compliance, out of the 343 patients, about 70.6% of them were compliant (Table 3).

**Table 1:** Socio-demographic characteristics of respondents

Variable	Frequency	Percentage (%)
*Age*		
20–30	26	7.6
31–50	85	24.8
51–60	87	25.4
> 60	145	42.3
*Gender*		
Male	129	37.6
Female	214	62.4
*Religion*		
Christianity	120	35
Islam	222	64.7
ATR	1	0.3
Ethnicity		
Dagomba	156	45.5
Gonja	59	17.2
Mamprusi	44	12.8
Frafra	19	5.5
Others	65	19
*Marital status*		
Single	34	9.9
Married	199	58
Divorced	23	6.7
Widowed	77	22.4
Separated	10	2.9
*Educational level*		
None	123	35.9
Primary	45	13.1
Middle/JHS	38	11.1
SHS/Vocational	70	20.4
Tertiary	67	19.5
*Occupational status*		
Employed	76	22.2
Unemployed	117	34.1
Self-employed	150	43.7
*Monthly Income*		
< GHȻ500	178	51.9
GHȻ500–GHȻ1000	81	23.6
> GHȻ1000	84	24.5

**Table 2:** Lifestyle and clinical characteristics of respondents

Variable	Frequency	Percentage (%)
*Smoking status*		
I smoke	13	3.8
I used to smoke	39	11.4
I have never smoked	291	84.8
*Alcohol consumption status*		
I take alcohol	27	7.9
I used to take alcohol	56	16.3
I have never taken alcohol	260	75.8
*Diabetes duration*		
< 5 years	143	41.7
5–10 years	94	27.4
> 10 years	106	30.9
*Medical Co-morbidity*		
Yes	209	60.9
No	134	39.1
*Nutritional knowledge*		
Good knowledge	160	46.6
Poor knowledge	183	53.4

**Table 3:** Adherence to dietary recommendations

Variable	Frequency	Percentage
*Do you sometimes forget to follow the recommended dietary approach for diabetes mellitus?*		
Yes	146	42.6
No	197	57.4
*Over the past two (2) weeks, were there any days when you didn’t follow your dietary plan properly?*		
Yes	107	31.2
No	236	68.8
*Did you miss the proper dietary plan yesterday?*		
Yes	64	18.7
No	279	81.3
*Have you ever cut back or stopped following the recommended dietary plan without telling your doctor because you felt unnecessary to do so?*		
Yes	121	35.3
No	222	64.7
*When you feel like your DM is under control, do you sometimes stop following your dietary plan?*		
Yes	124	36.2
No	219	63.8
*When you travel or leave home, are you sometimes forced to stop following your dietary plan?*		
Yes	127	37
No	216	63
*Do you ever feel hassled about sticking to dietary plan?*		
Yes	143	41.7
No	200	58.3
*Did you have feelings of dietary deprivation?*		
Yes	151	44
No	192	56
*Do you forget to include fruits and vegetables in your dietary plan?*		
Yes	99	28.9
No	244	71.1
*Do you forget to cut down butter and fat intake in your food?*		
Yes	66	19.2
No	277	80.8
*Overall compliance*		
Compliant	242	70.6
Not compliant	101	29.4

### Factors associated with dietary compliance

In the bivariate analysis, age (*P* < 0.001), marital status (*P* = 0.005), educational level (*P* = 0.006), smoking status (*P* = 0.006), alcohol consumption (*P* = 0.008), diabetes duration (*P* = 0.104), body mass index (*P* = 0.085) had *P*-values < 0.25 (Table 4), hence were considered for multivariate logistic regression analysis. In the multivariate analysis, age and educational level were identified as significant predictors of dietary compliance among the patients. As a patient’s age increases, compliance also decreases [Adjusted odd ratio (AOR): 0.964, 95% CI 0.941–0.987, *P* = 0.002]. Patients who completed JHS/Middle school were 2.4 times more likely to comply with dietary recommendations compared to those with no formal education [AOR: 2.458, 95% CI 1.019–5.928, *P* = 0.045]. Also participants who completed SHS/Vocational school were 2.7 times more likely to comply with dietary recommendations compared to those with no formal education [AOR: 2.725, 95% CI 1.075–6.908, *P* = 0.035] (Table 5).

**Table 4:** Bivariate analysis of factors associated with dietary compliance

Characteristic	Total population (n = 343)	Compliant (n = 242)	Non-compliant (n = 101)	P-Value
	Mean (SD)	Mean (SD)	Mean (SD)	
	n (%)	n (%)	n (%)	
Age (years)	1.29 (0.45)	58.91 (14.70)	50.50 (16.59)	< 0.001
*Gender*				
Male	129 (100)	84 (65.1)	45 (34.9)	0.26
Female	214 (100)	158 (73.8)	56 (26.2)	
*Marital status*				
Single	34 (100)	19 (55.9)	15 (44.1)	0.005
Divorced	23 (100)	12 (52.2)	11 (47.8)	
Separated	10 (100)	8 (80)	2 (20)	
*Ethnicity*				
Dagomba	156 (100)	111 (71.2)	45 (28.8)	0.65
Gonja	59 (100)	43 (72.9)	16 (27.1)	
Mamprusi	44 (100)	29 (65.9)	15 (34.1)	
Frafra	19 (100)	11 (57.9)	8 (42.1)	
Others	65 (100)	48 (73.8)	26.2 (16.8)	
*Educational level*				
None	123 (100)	97 (78.9)	26 (21.1)	0.006
Primary	45 (100)	26 (57.8)	19 (42.2)	
Middle/JHS	38 (100)	20 (52.6)	18 (47.4)	
SHS/vocational	70 (100)	48 (68.6)	22 (31.4)	
Tertiary	67 (100)	51 (76.1)	16 (23.9)	
*Occupational status*				
Employed	76 (100)	49 (64.5)	27 (35.5)	0.27
Unemployed	117 (100)	88 (75.2)	29 (24)	
Self-employed	150 (100)	105 (70.0)	45 (30)	
*Monthly income*				
Less than GHȻ500	178 (100)	125 (70.2)	53 (29.8)	0.70
GHȻ 500-GHȻ 1000	81 (100)	55 (67.9)	26 (32.1)	
Above GHȻ 1000	84 (100)	62 (73.8)	22 (26.2)	
*Smoking status*				
I smoke	13 (100)	4 (30.8)	9 (69.2)	0.006
I used to smoke	39 (100)	28 (71.8)	11 (28.2)	
I have never smoked	291 (100)	210 (72.2)	81 (27.8)	
*Alcoholic status*				
I take alcohol	27 (100)	12 (44.4)	15 (55.6)	0.008
I used to take alcohol	56 (100)	42 (75.0)	14 (25.0)	
I have never taken alcohol	260 (100)	185 (72.3)	72 (27.7)	
*Diabetes Duration*				
Less than 5 years	143 (1000	97 (67.8)	46 (32.2)	0.10
5–10 years	93 (100)	62 (60.0)	32 (34.0)	
Greater than 10 years	106 (100)	83 (78.3)	23 (21.7)	
*Medical co-morbidities*				
Yes	209 (100)	152 (72.7)	57 (27.3)	0.27
No	134 (100)	90 (67.2)	44 (32.8)	
*Diabetes nutritional education*				
Yes	336 (100)	238 (70.8)	98 (29.2)	0.43
No	7 (100)	4 (57.1)	3 (42.9)	
*Body mass Index*				
Underweight	17 (100)	8 (47.1)	9 (52.9)	0.08
Normal	191 (100)	136 (71.2)	55 (28.8)	
Overweight	102 (100)	71 (69.1)	31 (30.4)	
Obese	33 (100)	27 (81.8)	6 (18.2)	
*Nutritional knowledge*				
Good knowledge	160(100)	115(71.9)	45(28.1)	0.61
Poor knowledge	183(100)	127(69.4)	56(30.6)

**Table 5:** Multivariate analysis of factors associated with dietary compliance

Characteristics	Adjusted Odds Ratio (95% confidence interval)	*P*-value
*Age of patient*		
Age (years)	0.964 (0.941–0.987)	0.002
*Marital Status*		
Single	1	
Married	1.236 (0.191–7.997)	0.82
Divorced	1.612 (0.306–8.507)	0.57
Widow/Widower	3.455 (0.532–22.441)	0.19
Separated	1.019 (0.174–5.900)	0.98
*Educational status*		
None	1	
Primary	1.192 (0.533–2.605)	0.66
Middle/JHS	2.458 (1.019–5.928)	0.04
SHS/Vocational	2.725 (1.075–6.908)	0.03
Tertiary	1.459 (0.639–3.333)	0.37
*Smoking status*		
I smoke	1	
I used to smoke	3.022 (0.757–12.059)	0.11
I have never smoke	0.691 (0.284–1.684)	0.41
*Alcohol status*		
I take alcohol	1	
I used to take alcohol	2.469 (0.980–6.855)	0.05
I have never taken alcohol	0.868 (0.314–1.915)	0.72
*Diabetes duration*		
Less than 5 years	1	
5–10 Years	0.641 (0.298–1.379)	0.25
Greater than 10 years	1.138 (0.563–2.299)	0.71
*Body mass index*		
Underweight	1	
Normal weight	0.509 (0.156–1.659)	0.26
Overweight	0.584 (0.169–2.015)	0.39
Obese	0.444 (0.099–1.992)	0.28

## Discussion

Prevalence of type 2 diabetes is high worldwide and still increasing in every country including Ghana. Dietary compliance is critical for improving diabetes patients’ health outcomes. Hence the study examined dietary compliance and its associated predictors among Type-2 diabetics in the Tamale Metropolis of Ghana.

The present study revealed that 70.6% of the patients complied with the dietary recommendations. This finding is comparable to that of Ansah et al. [10] and Mugo et al. [14], in Ghana (69%) and Kenya (60%) respectively. Contrarily, the finding of the present study is higher than that of Wornyoh [12], Abate et al. [13], Baral et al. [15] and Alhariri et al. [16] in Ghana (26.7%), Ethiopia (41.1%), Nepal (15.7%) and Yemen (21%) respectively. The studies vary in terms of dietary compliance assessment tools, population characteristics, sample size and geographical location, hence the differences in findings [23, 24].

In the present study, age was identified as a predictor of dietary compliance. As age of patient increases, dietary compliance decreases. Majority of elderly people experience memory issues and diminished cognitive performance, as such, following a dietary regimen becomes a challenge [25]. Similarly, studies by Parajuli et al. [25], Salam and Siddiqui [26], Alhariri et al. [16], Mirahmadizadeh et al. [27] and Anderson and Gustafson [28] revealed that compliance to dietary recommendations declines with increasing age among Type 2 diabetes patients.

It was also observed in the present study that higher level of education was positively related to dietary compliance as patients who completed JHS and SHS were more likely to comply to the recommended diet as compared to those with no formal education. Likewise, a study by Demilew et al. [29] reported a significant positive relationship between higher level of education and dietary compliance among type 2 diabetes patients. Studies by Patel et al. [30] and Al-Rasheedi [31] also revealed that patients who adhere to recommended diet have higher level of education. The reason for this could be related to the impact of higher educational level on patients’ adherence to dieticians’ advice [32]. In the present study, gender, marital status, smoking, alcoholism, duration of diabetes and body mass index did not show statistically significant relationship with dietary compliance in the multivariate analysis. In consonance with the findings of the present study, a study by Alhariri et al. [16] to determine the factors associated with adherence to diet among type 2 diabetes patients, revealed that gender, marital status, smoking and body mass index are not correlated with dietary compliance. Similarly, Parajuli et al. [20] found no association between diabetes duration and dietary adherence. On the other hand, these factors have shown to be associated with dietary compliance among type 2 diabetes patients in other studies [17, 26, 27, 33]. The differences in findings among the studies may be attributed to differences in methodology and population characteristics [23].

The findings of this study should be evaluated in light of the following inescapable limitations, despite great attempts to minimize any potential shortcomings of study. The cross-sectional nature of the study did not offer a solid foundation for determining causation. Given that the study was self-reported, response bias may have impacted the precise estimation of dietary adherence.

## Conclusion

The rate of compliance to dietary recommendations was high among participants in the study hospitals. The study also showed that age and educational status significantly influenced dietary compliance, suggesting that these factors should be taken into account when developing strategies to enhance dietary adherence.

## Data Availability

The datasets used and/or analysed during the current study are available from the corresponding author on reasonable request.

## Abbreviations

AOR: Adjusted odd ratio
CI: Confidence interval
SHS: Senior high school
JHS: Junior high school
SPSS: Statistical package for social sciences
Kg: Kilogram
M: Metres
IBM: International business machines
USA: United States of America
